# Supplementary material for: New Insights into Avian Infectious Bronchitis Virus in Colombia from Whole-Genome Analysis
Source: Viruses. 2022 Nov 19;14(11):2562. doi: 10.3390/v14112562 (PMC9694374; doi:10.3390/v14112562)
Supplement: Supplementary file 1 [file viruses-14-02562-s001.zip › viruses-1984736-supplementary.pdf]

Table S1. Read filtering and assembly statistics for IBV isolates included in the study.

Sample	Paired Reads	% Clean Reads	% Reads that generated IBV contigs	Final assembly Size (nt)
V2	2,108,222	75.7	66.84	25,531
V3	985,232	96.1	81.98	27,625
V5	1,956,368	87.1	44.35	24,102
V6	2,027,026	86.3	7.86	27,532
V8	2,451,922	77.5	88.37	27,559
V9	1,070,220	93.8	85.78	25,783
V10	1,261,698	92.2	38.85	27,558
**Average**	**1,694,384**	**87.0**	**59.15**	**26,527**
